# The exocyst and regulatory GTPases in urinary exosomes

**DOI:** 10.14814/phy2.12116

**Published:** 2014-08-19

**Authors:** Maria F. Chacon‐Heszele, Soo Young Choi, Xiaofeng Zuo, Jeong‐In Baek, Chris Ward, Joshua H. Lipschutz

**Affiliations:** 1 Renal, Electrolyte and Hypertension Division, Department of Medicine, University of Pennsylvania, Philadelphia, Pennsylvania; 2 Renal Division, Department of Medicine, Medical University of South Carolina, Charleston, South Carolina; 3 Division of Nephrology and Hypertension, Department of Internal Medicine, University of Kansas Medical Center, Kansas City, Kansas; 4 Department of Medicine, Ralph H. Johnson VAMC, Charleston, South Carolina

**Keywords:** Cilia, ciliopathies, ELV, exocyst, polycystic kidney disease, urinary exosomes

## Abstract

Cilia, organelles that function as cellular antennae, are central to the pathogenesis of “ciliopathies”, including various forms of polycystic kidney disease (PKD). To date, however, the molecular mechanisms controlling ciliogenesis and ciliary function remain incompletely understood. A recently proposed model of cell–cell communication, called “urocrine signaling”, hypothesizes that a subset of membrane bound vesicles that are secreted into the urinary stream (termed exosome‐like vesicles, or ELVs), carry cilia‐specific proteins as cargo, interact with primary cilia, and affect downstream cellular functions. This study was undertaken to determine the role of the exocyst, a highly conserved eight‐protein trafficking complex, in the secretion and/or retrieval of ELVs. We used Madin–Darby canine kidney (MDCK) cells expressing either Sec10‐myc (a central component of the exocyst complex) or Smoothened‐YFP (a ciliary protein found in ELVs) in experiments utilizing electron gold microscopy and live fluorescent microscopy, respectively. Additionally, human urinary exosomes were isolated via ultracentrifugation and subjected to mass‐spectrometry‐based proteomics analysis to determine the composition of ELVs. We found, as determined by EM, that the exocyst localizes to primary cilia, and is present in vesicles attached to the cilium. Furthermore, the entire exocyst complex, as well as most of its known regulatory GTPases, are present in *human* urinary ELVs. Finally, in living MDCK cells, ELVs appear to interact with primary cilia using spinning disc confocal microscopy. These data suggest that the exocyst complex, in addition to its role in ciliogenesis, is centrally involved in the secretion and/or retrieval of urinary ELVs.

## Abstract (precis)

Our data suggest that the exocyst complex, in addition to its role in ciliogenesis that we previously described, is centrally involved in the secretion and/or retrieval of urinary exosomes. These results could have important implications for PKD, other renal diseases, as well as normal kidney homeostasis.

## Introduction

Primary cilia, found at the surface of many cells, are sensory organelles known to perceive protein‐mediated (e.g. Hedgehog) and mechanical signals (e.g. fluid flow). Defects in ciliary function lead to a number of human diseases, termed ciliopathies. Ciliopathies can affect the kidney, where mutations that lead to disruption of ciliary form or function result in polycystic kidney disease (PKD). For example, autosomal dominant and recessive PKD (ADPKD and ARPKD), and nephronopthisis, are ciliopathies caused by mutations in genes encoding the ciliary proteins polycystin‐1 (Consortium 1995; Hughes et al. 1995; Yoder et al. 2002) (PC‐1, ADPKD), PC‐2 (Mochizuki et al. 1996; Yoder et al. 2002; Boletta and Germino 2003) (ADPKD), fibrocystin (Zerres et al. 1994; Onuchic et al. 2002; Ward et al. 2002, 2003) (ARPKD), and the nephrocystins (nephronophthisis) (Jauregui 2007; Hurd and Hildebrandt 2010). The cystic overgrowth characteristic of PKD ultimately leads to the destruction of kidney architecture and renal failure (Grantham 2001). Although PKD accounts for ~5% of all cases of end‐stage kidney disease (ESKD) (Kc and Ly 2002; Ibraghimov‐Beskrovnaya and Bukanov 2008) in the United States and, thus presents a significant burden, the molecular mechanisms linking ciliary mutations to the cystic phenotype remain unclear. Given that supportive care is the only “treatment” currently approved for any form of PKD, a better understanding of ciliary biology is essential to develop much‐needed new therapies for PKD. Here, we examine a novel signaling mechanism that links renal primary cilia to exosomes and the exocyst complex.

Exosomes are small, membrane‐bound vesicles that are released into the extracellular environment (Mathivanan et al. 2010). Exosomes can mediate cell–cell communication and affect signal transduction in recipient cells in both normal and pathological conditions (Calzolari et al. 2006; Admyre et al. 2007; Mallegol et al. 2007; Masyuk et al. 2010; Lee et al. 2012; Li et al. 2012). For example, platelet‐derived exosomes regulate coating events (Cocucci et al. 2009); exosomes from intestinal epithelia activate the mucosal system (Mallegol et al. 2007); while tumor‐derived exosomes transfer oncogenic receptors to receiving cells (Al‐Nedawi et al. 2008). In the kidney, and other organs, exosomes have been suggested to carry disease‐specific biomarkers (e.g. for acute kidney injury, chronic kidney disease, podocyte injury, cancers, and PKD (Pisitkun et al. 2006; Cheruvanky et al. 2007; Camici 2008; D'Souza‐Schorey and Clancy 2012)). Lending credence to this idea, a very recent study showed that specific *C. elegans* ciliated sensory neurons shed and release exosome‐sized extracellular vesicles containing GFP‐tagged polycystins LOV‐1, the PKD‐1 *C. elegans* ortholog (Barr and Sternberg 1999), and PKD‐2 and that these extracellular vesicles were abundant in the lumen surrounding the cilium (Wang et al. 2014). Furthermore, EM and genetic analysis indicated that the extracellular vesicle biogenesis occurred via budding from the plasma membrane at the ciliary base, and not via fusion of multivesicular bodies, and that intraflagellar transport and kinesin‐3 KLP‐6 were required for environmental release of PKD‐2::GFP‐containing extracellular vesicles. Finally, extracellular vesicles isolated from wild‐type animals induced male tail‐chasing behavior, while extracellular vesicles isolated from klp‐6 animals and lacking PKD‐2::GFP did not, indicating that environmentally released extracellular vesicles play a role in animal communication and mating‐related behaviors (Wang et al. 2014). Given the growing evidence of the existence and biological importance of these cilia‐interacting vesicles, the question of how they're regulated within the cell arises. We hypothesize that the exocyst complex plays a critical role in regulating these vesicles.

The exocyst complex (a.k.a. the exocyst) is a ~750 kDa octameric protein complex initially identified in *S. cerevisiae* and highly conserved from yeast to mammals (Novick et al. 1980; Hsu et al. 1996). The mammalian exocyst comprises Exoc1/Sec3, Exoc2/Sec5, Exoc3/Sec6, Exoc4/Sec8, Exoc5/Sec10, Exoc6/Sec15, Exoc7/Exo70, and Exoc8/Exo84 (Novick et al. 1980; Hsu et al. 1996) and is best known for its role in targeting and docking vesicles carrying membrane proteins from the trans‐Golgi network (TGN) (Lipschutz and Mostov 2002). Importantly, we previously showed, in renal tubule cells, that exocyst components are localized to primary cilia (Rogers et al. 2004), that the exocyst is required for ciliogenesis (Zuo et al. 2009), and that the exocyst genetically interacts with polycystin‐2 in zebrafish (Fogelgren et al. 2011; Choi et al. 2013). Mutations in an exocyst component were also recently shown to cause PKD in a human family with Joubert Syndrome, a nephronophthisis form of PKD (Dixon‐Salazar et al. 2012). Thus, a link between the exocyst complex, primary cilia, and cystic kidney disease, has been established. We have also shown, via electron microscopy (EM) (Hogan et al. 2009; Bakeberg et al. 2011), that cilia appear to interact with exosomes. Exosome‐like vesicles (ELVs) are a subset of exosomes that carry *cilia‐specific* membrane proteins, including various proteins involved in polycystic kidney disease, such as PC‐2, as well as other ciliary membrane proteins, such as Smoothened (Smo) (Hogan et al. 2009; Bakeberg et al. 2011).

Given that understanding the mechanisms that mediate cilia/ELV interactions could be critical for understanding the biology that links cilia to renal pathogenesis, particularly to different forms of PKD, we decided to further explore a possible link between renal primary cilia, urinary ELVs, and the exocyst complex. Here, we show via transmission electron microscopy (TEM) with immunogold labeling that the exocyst complex localizes to both primary cilia in renal epithelial cells, and to membrane‐vesicles that interact with primary cilia. We further confirm this finding by showing that *every member of the exocyst complex,* as well as most of its known regulatory GTPases, is present in purified human urinary ELVs. Lastly, we expand on previous EM work to show that exosomes appear to interact with primary cilia in renal cells. Taken together, these data support the idea of a functional/physical link exists between primary cilia, ELVs, and the exocyst complex. Furthermore, given the previously established relationship between cilia, the exocyst complex, and renal cystogenesis, we hypothesize that not only is the exocyst complex at the right place to mediate cilary/ELV interactions, but that these processes play a central role in the biology of PKD.

## Materials and Methods

### Cell culture

Type II Madin–Darby canine kidney (MDCK) cells were used between passages 3 and 10. These cells were originally cloned by Dr. D. Louvard (European Molecular Biology Laboratory, Heidelberg, Germany) and came to us via Dr. Mostov who obtained them from Dr. K. Matlin (University of Chicago, Chicago, IL). We previously generated MDCK type II cells overexpressing myc‐tagged‐hSec10 (Lipschutz et al. 2000). MDCK type II cells overexpressing Smoothened‐YFP were generously provided to us by Drs. Ott and Lippincott‐Schwartz (Ott et al. 2012). Cells were cultured in modified Eagle's minimal essential medium containing Earl's balanced salt solution and glutamine supplemented with 5% fetal calf serum, 100 U/mL penicillin, and 100 μg/mL streptomycin. MDCK cells were seeded at confluence on 24‐mm Transwell filter units coated with collagen (Corning Life Sciences, Lowell, MA). Cell monolayers were used for experiments after 7–14 days of culture with daily changes in medium.

### Transmission electron microscopy

MDCK cells were grown on Transwell filters for 7 days and subsequently fixed in a solution containing 2% glutaraldehyde, 0.8% paraformaldehyde, and 0.1 M cacodylate. Cells were stained with osmium and imidazole as described previously (Lipschutz et al. 2000). Briefly, cells were dehydrated, embedded in resin, and sectioned. Immunolabeling was performed using mouse anti‐myc antibody (mouse monoclonal anti‐myc, **#**2276 Cell Signaling, at 1:200 dilution) to identify the myc epitope present on Sec10. Secondary goat anti‐mouse antibody (Jackson ImmunoResearch, West Grove, PA) was tagged with ultrasmall gold (Aurion, Immuno Gold Reagents & Accessories, Wageningen, the Netherlands). The gold label was further enhanced with silver staining for 25 minutes. Finally, the cells were imaged on a FEI Tecnai transmission electron microscope (JEOL 1010) fitted with a Hamamatsu digital camera and imaging software from Advanced Microscopy Techniques, Danvers, MA.

### Spinning disk confocal microscopy

Smoothened‐YFP MDCK cells were grown upside‐down on Transwell filters for 7 days. To accomplish this, Smoothened‐YFP MDCK cells were seeded on the bottom of inverted Transwell filters, allowed to attach, and the Transwell filters were then placed in the normal upright position for 7 days. For imaging, Transwell filters, containing the cells growing on the bottom of the filters, were placed on glass‐bottom cultured plates with the apical cell surface (where the cilia are located) touching the glass. This allowed for optimal imaging. Cells were imaged at the University of Pennsylvania CDB microscopy Core using an Olympus IX71 spinning disk confocal microscope fitted with a Hamamatsu ImagEM EMCCD camera and Metamorph imaging software. Images were processed in Fiji (Image J) Software to add scale bar and time stamps, and to generate the time‐lapse illustrations of ELV/cilia interactions. The labels and arrows were added to the time‐lapse panels in Adobe Photoshop.

### Isolation of human urinary ELVs and mass spectrometry analysis

Urinary ELVs were isolated following a modification of the methods of Pisitkun et al. (2004) and Zhou et al. (2006) as previously described (Hogan et al. 2009; Chen et al. 2013). Briefly, the first void of the day was collected and treated with one tablet of Complete Protease Inhibitor, EDTA‐free (Roche, New York, NY) to continuously inhibit serine and cysteine proteases. If the samples were not to be used immediately, the urine was frozen at −80°C, and then vigorously vortexed during thawing when it was ready to be used. To pellet cellular debris, the samples were subsequently centrifuged at room temperature in a Sorvall Revolution RC centrifuge 4,275‐rpm (4,000 × *g*) using at SLC‐600 rotor. The supernatant was then filtered using an 80‐*μ*m nylon mesh filter to prevent the carryover of any pelleted material into subsequent centrifugation steps. The filtered supernatant was subsequently centrifuged at 45,000 rpm (225,000 × *g*) for 2 h at 4°C in a Sorvall Discovery 90SE ultracentrifuge to generate a “crude ELV” fraction containing Tamm–Horsfall protein (THP) and a few ELVs. To remove THP, crude ELV fractions were layered on top of a 5–30% sucrose gradient with heavy water (D_2_O). The gradient was centrifuged at 200,000 × *g* for 24 h, and then the fractions were harvested, diluted five‐ to 10‐fold in PBS and centrifuged at 150,000 × *g* for 1 h to recover PKD‐ELVs. These were stored in 100 μL of 0.25 M Sucrose 20 mM HEPES (pH 6.0) with 1× Complete Protease Inhibitor and frozen down at −80°C if they were not immediately used. Purified ELV fractions were run on MOPS gels in normal water, stained and destained, and digested with trypsin again in normal water, diluting residual D_2_O to background levels before proteomic analysis, and reversing any potential exchange reactions. The total time exposed to normal water was at least 5 days. Subsequently, PKD‐ELV fractions were subjected to mass spectrometry analysis to identify proteins present in ELVs (Hogan et al. 2009; Chen et al. 2013).

### Proteomics analysis of the exocyst

We searched our ELV proteome for the exocyst complex as well as GTPases using Scaffold Software (Proteome Software, Inc., Portland, OR). All members of the exocyst complex were quickly identified using the search term “EXOC”. To identity GTPases in the ELV proteome, we used the following search terms “Arf”, “Arl”, “Cdc”, “GTP”, “Rab”, “Rac”, “Ral”, “Ras” and “Rho”. Lists containing any of these terms were exported to Microsoft Excel where duplicate results were removed. Known GTPases within this group, were identified using functional cluster analysis on DAVID Bioinformatics Resources 6.7 (Huang et al. 2009) yielding over 100 potential GPTase or GTPase regulatory proteins present in ELVs.

To determine which, if any, of these proteins were previously known to interact with the exocyst, a gene interaction network was established by searching Biogrid 3.2 (a literature‐curated database of protein and genetic interactions, www.thebiogrid.org) (Stark et al. 2006) and/or GeneMania database (www.genenamia.org, University of Toronto, Toronto, Canada) (Montojo et al. 2010) for each exocyst component. These data were used to create the network diagram limited to the GTPases *currently known* to interact with the exocyst complex using Cytoscape 3.2 Software (www.cytoscape.org) (Cline et al. 2007; Saito et al. 2012) (Fig. 3). This list was manually compared to the entire ELV GTPase list, where we found all nine mammalian exocyst regulatory GTPases identified through the aforementioned literature‐curated databases. The aforementioned tools were also utilized to build the first‐neighbor interaction diagrams for Smoothened (Fig. 4B) and for Polycystins 1 and 2, and Fibrocystin (Fig. 4C).

### Human participants

As previously reported (Hogan et al. 2009), urine samples were obtained from normal human volunteers. This study was performed in adherence to the Declaration of Helsinki and approved by the Mayo Clinic IRB:09003355: “Analysis of the proteome of PKD‐ELVs in polycystic kidney disease and controls”.

## Results

### The exocyst localizes to primary cilia and cilia‐interacting vesicles

**Figure 1.**
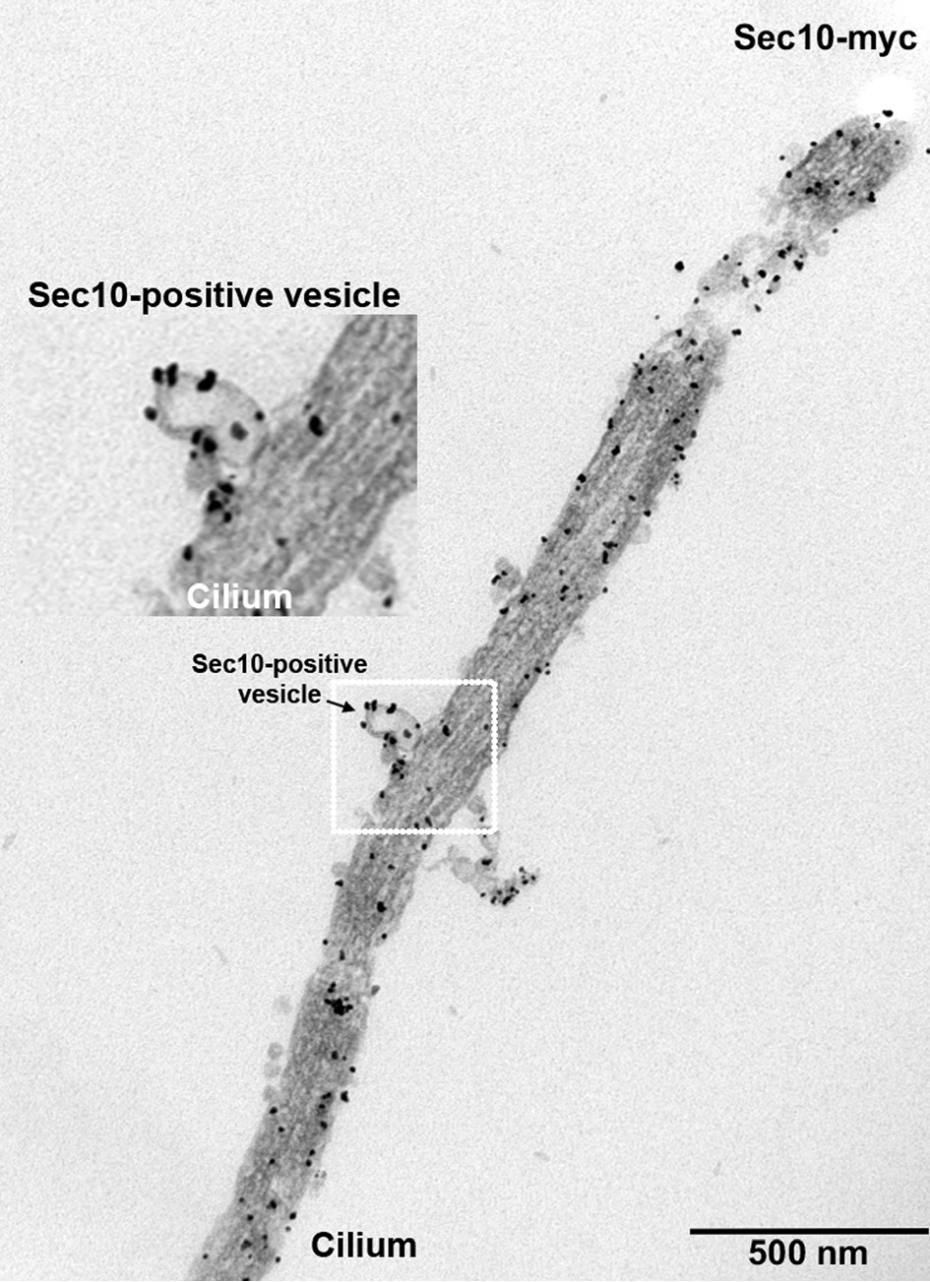
The exocyst protein Sec10 localizes to primary cilia in MDCK cells. TEM analysis, with immunogold labeling of the myc antibody, in MDCK cells expressing hSec10‐myc shows that this essential member of the exocyst complex localizes along the length of the primary cilium and is found in membrane‐bound vesicles interacting with primary cilia (inset). Control MDCK cells showed few, if any, gold particles localizing to the cell or the primary cilium (data not shown).

To determine the localization of the exocyst complex, we used MDCK cells that stably express myc‐tagged‐human Sec10 (hSec10‐myc cells) (Lipschutz et al. 2000). To visualize the intracellular localization of hSec10, Transwell filter‐grown hSec10‐myc MDCK‐II cells were fixed and subjected to transmission electron microscopy (TEM). Since an anti‐hSec10‐antibody suitable for EM was not available, we used a mouse anti‐myc monoclonal antibody, and a silver enhanced gold secondary antibody to visualize myc‐tagged hSec10 (Zuo et al. 2009). Here, we confirm that the exocyst component Sec10 is localized to renal primary cilia in MDCK cells (Fig. 1). Moreover, we show that in addition to being present at the base of the primary cilium, where it would be expected, based on the known role of the exocyst in vesicle trafficking, exocyst Sec10 was found all along the primary cilium, and in what appear to be membrane bound vesicles interacting with the primary cilium (Fig. 1, insets). Given that Sec10 is an essential member of the exocyst complex (Zuo et al. 2009), these data suggest that the exocyst is present in membrane vesicles that interact with primary cilia.

### The exocyst is present in cilia‐interacting exosome‐like vesicles (ELVs)

**Figure 2.**
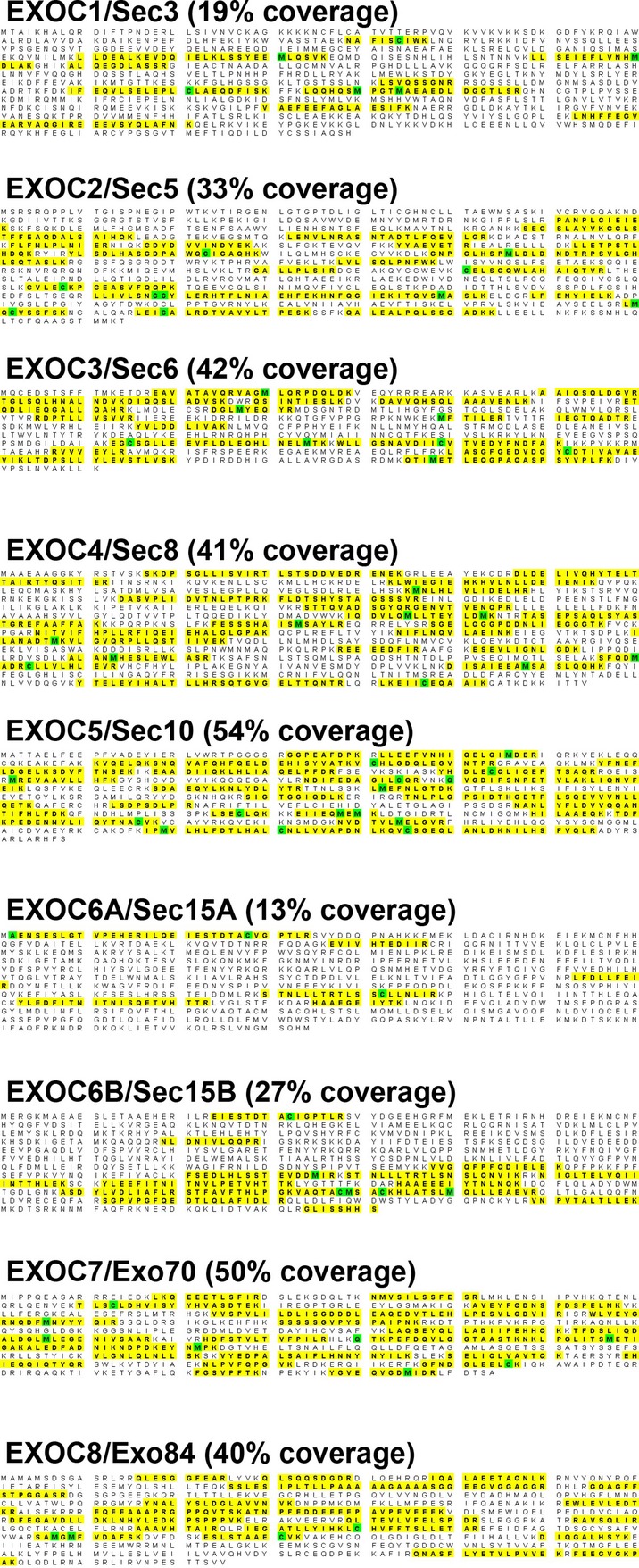
ELVs contain all eight members of the exocyst complex. Proteomic analysis of ELVs allowed for identification of all eight members of the exocyst complex. Schematic diagrams are shown illustrating the different unique peptides identified for each member of the exocyst (highlighted). Please note that the protein sequence identified (% coverage) for each exocyst component ranged from 13% (e.g. Exo6A/Sec15B) to 54% (e.g. ExoC5/Sec10), which is considered significant. The green highlighting indicates modification of the residue. The variable (variable meaning they were allowed to be present or absent) modifications searched for were as follows: (1) Carbamidomethylation of cysteine (the expected modification from alkylating cysteines with iodoacetamide after reducing with DTT). (2) Propionamide modification on cysteine (originates from free acrylamide present in the gels reacting with cysteines). (3) Oxidation of methionine‐ can originate biologically, but more predominantly present from oxidation during sample handling, while running gel, and/or from oxidation during the electrospray process during LC‐MS/MS.

To determine whether the exocyst complex is found in ELVs, we purified secreted vesicles from human urine. Briefly, human urine was subjected to ultracentrifugation to generate a crude exocyst preparation, which was subsequently fractionated using a D_2_O sucrose gradient to generate a fraction enriched for a subset of exosomes that carry cilia‐specific proteins such as polycystin‐2, which we have previously named exosome‐like vesicles or ELVs (Hogan et al. 2009). ELV‐rich fractions were subjected to mass‐spectrometry‐based proteomic analysis to identify ELV cargo as previously described (Hogan et al. 2009). Supporting our findings from TEM, analysis of *human* urinary exosomes shows that all eight members *of the exocyst complex* are found in urinary ELVs (Fig. 2). This proteomic analysis shows multiple *unique* peptides identified for each protein (highlighted for each protein) covering from 20% to more than 50% for each of the eight component proteins of the exocyst complex. Importantly, *all eight members of the mammalian exocyst complex* were present in the ELVs suggesting that the entire complex, and not just individual exocyst proteins, have a function in ELV biology.

### Small GTPases that regulate the Exocyst are present in ELVs

**Figure 3.**
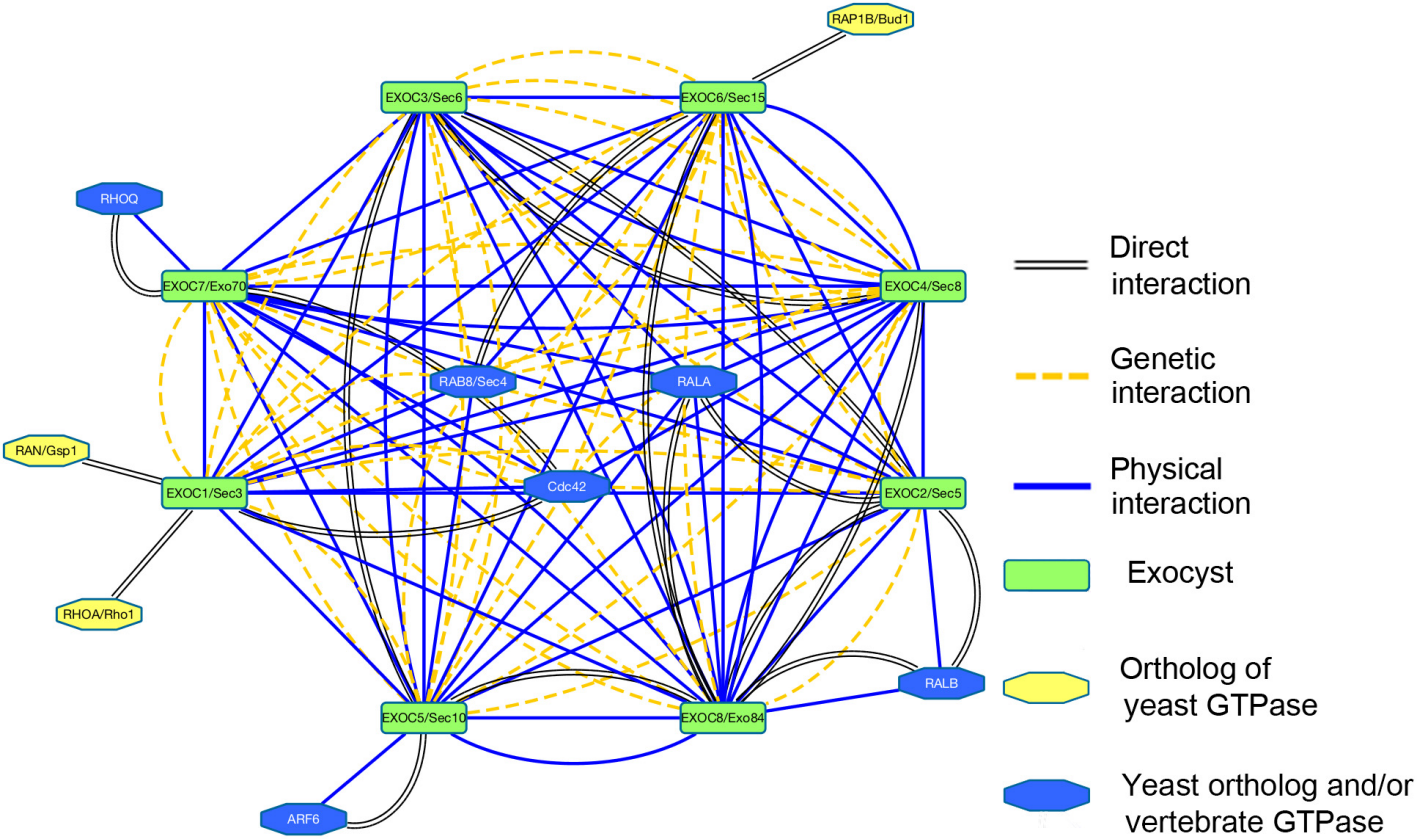
ELVs carry most of the small GTPases known to regulate the exocyst complex. Schematic diagram illustrating the interactions between the different exocyst subunits and the nine mammalian GTPases, known to regulate the exocyst complex that we found in human urinary ELVs. “Ortholog of yeast GTPase” refers to mammalian orthologs of yeast GTPases that interact with the exocyst. “Yeast ortholog and/or vertebrate GTPase”, refers to mammalian GTPases that interact with the exocyst (the yeast ortholog of Rab8 is Sec4, and both Rab8 and Sec4 have been shown to interact with the exocyst, so that is included here as well).

To determine if the exocyst was just a “bystander” or has a more functional role in ELVs, we searched for GTPases that have been shown to regulate the exocyst. We found that, among many other GTPases, GEFs and GAPS, ELVs carry nine small GTPases known to physically interact with and/or regulate the exocyst (Fig. 3, Table 1), including: ARF6, which interacts with Exoc5/Sec10 (Prigent et al. 2003); RAL A, which interacts with Exoc2/Sec5 and Exoc8/Exo84 (Formstecher et al. 2005), RAL B which interacts with Exoc2/Sec5 and Exoc8/Exo84 (Moskalenko et al. 2003); RHOQ, which interacts with Exoc7/Sec15 (Inoue et al. 2003); RAN/GSP1, which interacts with Exoc1/Sec3 (Braunwarth et al. 2003), RHOA/RHO1 which interacts with Exoc1/Sec3 (Guo et al. 2001; Zhang et al. 2001; Yamashita et al. 2010); RAB8/SEC4 which interacts with Exoc1/Sec3 (Finger et al. 1998) and EXOC6/Sec15 (Bowser et al. 1992; Guo et al. 1999; Heger et al. 2011), RAP1B/Bud1 which interacts with Exoc6/Sec15 (Drees et al. 2001); and CDC42, which interacts with Exoc1/Sec3 and Exoc5/Sec10 (we showed this genetically in zebrafish, as well as by co‐IP) (Zhang et al. 2001, 2008; Wu and Brennwald 2010), and Exoc7/Exo70 (Wu and Brennwald 2010; Wu et al. 2010). Thus, ELVs contain the entire exocyst complex and most of its known regulatory GTPases.

**Table 1.** Exocyst regulatory GTPases present in human urinary ELVs.

Entrez ID	Name	Human Ortholog	Human Entrez ID	Present in ELVs
382	ARF6	ARF6	382	Yes
5898	RALA	RALA	5898	Yes
5899	RALB	RALB	5899	Yes
23433	RHOQ	RHOQ	23433	Yes
856294	RHO1	RHOA	387	Yes
850930	CDC42	CDC42	998	Yes
851000	GSP1p	RAN	5901	Yes
853056	RSR1	RAP1B	5908	Yes

### Small, fluorescently tagged membrane‐vesicles appear to interact with cilia in MDCK cells

**Figure 4.**
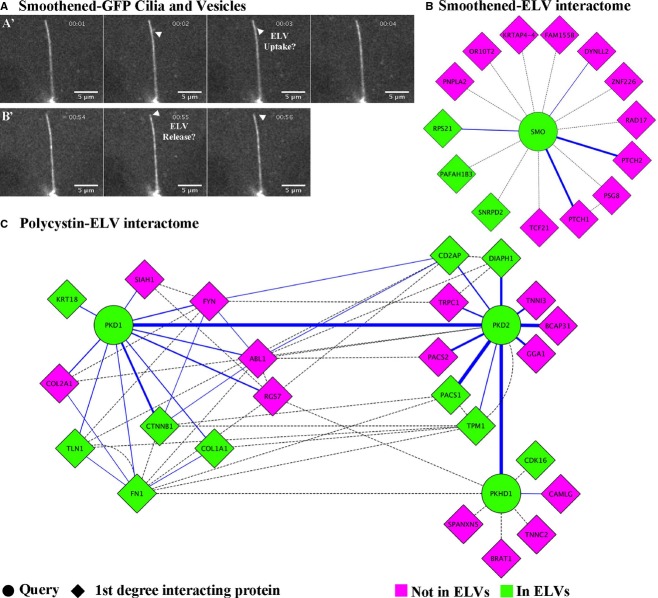
Fluorescently labeled ELVs appear to interact with renal primary cilia. (A) Still image from a spinning disk confocal movie (Supplemental movie 1) of Smoothened‐YFP MDCK cells showing small Smoothened‐containing vesicles (arrowheads) in the extracellular region interacting with renal primary cilia. A'. Small vesicles appear to interact with primary cilia. B'. A small vesicle appears to be released to the extracellular environment from the tip of the primary cilium. (B) First‐degree interaction map for Smoothened illustrating which proteins are present (green) and absent (magenta) in human ELVs. (C) First‐degree interaction map for PKD1 which encodes Polycystin‐1, PKD2 which encodes Polycystin‐2, and PKDH1 which encodes Fibrocystin, illustrating which proteins are present (green) and absent (magenta) in human ELVs. The solid blue line refers to a physical interaction that has been reported, and the dotted line refers to a genetic interaction.

To further examine a possible interaction between exosomes and the primary cilium, we used MDCK cells that express fluorescently tagged ciliary membrane protein Smoothened (Smoothened‐YFP/SmoYFP) (Ott et al. 2012), which is found in ELVs, and spinning disk and TIRF microscopy. We identified small vesicles that appear to interact with renal epithelial tubule cell primary cilia (Fig. 4A and Movie S1). However, given the fact that the size of the exosomes is very close to (or below) the resolution limits of light microscopy, further work will be required to capture these potential interactions at greater resolution, and to determine the functional roles of these processes in different cells.

### ELVs carry proteins known to interact with Smoothened, Polycystins 1 and 2 and Fibrocystin

We found that ELVs carry three out of 14 first‐neighbors/first‐degree Smoothened interactors (Fig. 4B). As described in Table 2, these are RPS21, which physically interacts with Smoothened; and PAFAH1B3 and SNRPD2, which are known to genetically interact with Smoothened.

**Table 2.** Smoothened interactors in human urinary ELVs.

Symbol	Protein name	Uniprot ID	Interaction type	Present in ELVs
TCF21	Transcription Factor 21	O43680	Genetic	No
RAD17	RAD 17 homolog	O75943	Genetic	No
FAM155B	Family with sequence similarity 155, member B	O75949	Physical	No
RPS21	Ribosomal protein S21	P08865	Physical	Yes
SNRPD2	Small nuclear ribonucleoprotein D2	P62316	Genetic	Yes
PTCH1	Patched 1	Q13635	Physical	No
PAFAH1B3	Platelet‐activating factor acetylhydrolase 1b	Q15102	Genetic	Yes
OR10T2	Olfactory receptor, family 10, subfamily T, member 2	Q8NGX3	Genetic	No
PNPLA2	Patatin‐like phospholipase domain containing 2	Q96AD5	Genetic	No
DYNLL2	Dynein, light chain, LC8‐type2	Q96FJ2	Physical	No
SMO	Smoothened	Q99835	^1^	Yes
KRTAP4‐4	Keratin associated protein 4‐4	Q9BYR3	Genetic	No
ZNF226	Zinc finger protein 226	Q9NYT6	Genetic	No
PSG8	Pregnancy specific beta‐1‐glycoprotein 8	Q9UQ74	Genetic	No
PTCH2	Patched 2	Q9Y6C5	Physical	No

^1^ Queried protein: “Queried protein” refers to the protein for which we searched the database for known interacting proteins that are found in ELVs.

Likewise, ELVs also carry proteins that interact with Polycystins 1 and 2 and Fibrocystin in a first‐degree basis (Fig. 4C). As illustrated in Fig. 4C, we found six out of 11 Polycystin‐1 interactors, six out of 13 polycystin‐2 interactors, and two out of seven Fibrocystin interactors in human urinary ELVs (Table 3). The Polycystin‐1 interactors were as follows: talin‐1 (TLN1); Polycystin‐2 (PKD‐2), beta catenin (CTNNB1); keratin‐18 (KRT‐18); fibronectin (FN1); and the alpha I chain of collagen (COL1A). Notably, all of these proteins are known to physically interact with Polycystin‐1 (Table 3). The Polycystin‐2 interactors were as follows: tropomyosin‐1 (TPM1); phosphofurin acidic cluster sorting protein 1 (PACS1); Fibrocystin (PKHD1); protein diaphanous homolog 1 (DIAPH1); cyclin‐dependent kinase 16 (CDK16); and fibronectin (FN1). With the exception of CDK16 and FN1, which interact genetically with PKD‐2, all of the above are known to physically interact with Polycystin‐2. Lastly, both Fibrocystin interactors identified in human urinary ELVs, namely CDK16 and FN1, genetically interact with PKHD1.

**Table 3.** PKD1, PKD2 and PKHD1 interactors in human urinary ELVs.

Symbol	Protein Name	Uniprot ID	Interaction Type	Interacts with	In ELVs
TLN1	Talin‐1	Q9Y490	Physical	PKD1	Yes
SIAH1	E3 Ub Ligase SIAH1 (seven in absentia homolog 1)	Q8IUQ4	Physical	PKD1	No
RGS7	Regulator of G‐protein signaling 7	P49802	Physical	PKD1	No
PKD2	Polycystin 2	Q13563	Physical	PKD1	Yes
CTNNB1	Beta Catenin	P35222	Physical	PKD1	Yes
FYN	Tyrosine‐protein kinase Fyn	P06241	Physical	PKD1	No
KRT‐18	Keratin, type I cytoskeletal 18	P05783	Physical	PKD1	Yes
FN1	Fibronectin	P02751	Physical	PKD1	Yes
COL2A1	Collagen alpha‐1(II) chain	P02458	Physical	PKD1	No
COL1A1	Collagen alpha‐1(I) chain	P02452	Physical	PKD1	Yes
ABL1	Abelson murine leukemia viral oncogene homolog 1	P00519	Physical	PKD1	No
TRPC1	Short transient receptor potential channel 1	P48995	Physical	PKD2	No
TPM1	Tropomyosin‐1	P09493	Physical	PKD2	Yes
TPM1	Tropomyosin‐1	P09493	Genetic	PKD2	Yes
TNNI3	Cardiac troponin I	P19429	Physical	PKD2	No
CD2AP	CD2‐associated protein	Q9Y5K6	Physical	PKD2	Yes
GGA1	ADP‐ribosylation factor‐binding protein GGA1	Q9UJY5	Physical	PKD2	No
PACS2	Phosphofurin acidic cluster sorting protein 2	Q86VP3	Physical	PKD2	No
PACS1	Phosphofurin acidic cluster sorting protein 1	Q6VY07	Physical	PKD2	Yes
PKHD1	Fibrocystin	P08F94	Physical	PKD2	Yes
BCAP31	B‐cell receptor‐associated protein 31	P51572	Physical	PKD2	No
COL2A1	Collagen alpha‐1(II) chain	P02458	Genetic	PKD2	No
ABL1	Abelson murine leukemia viral oncogene homolog 1	P00519	Genetic	PKD2	No
DIAPH1	Protein diaphanous homolog 1	O60610	Physical	PKD2	Yes
TNNC2	Troponin C, skeletal muscle	P02585	Genetic	PKHD1	No
SPANXN5	Sperm protein associated with the nucleus on the X chromosome N5	Q5 MJ07	Genetic	PKHD1	No
RGS7	Regulator of G‐protein signaling 7	P49802	Genetic	PKHD1	No
BRAT1	BRCA1‐associated ATM activator 1	Q6PJG6	Genetic	PKHD1	No
CDK16	Cyclin‐dependent kinase 16	Q00536	Genetic	PKHD1	Yes
CAMLG	Calcium signal‐modulating cyclophilin ligand	P49069	Physical	PKHD1	No
FN1	Fibronectin	P02751	Genetic	PKHD1	Yes
FN1	Fibronectin	P02751	Genetic	ABL1	Yes
TLN1	Talin‐1	Q9Y490	Genetic	CD2AP	Yes
FYN	Tyrosine‐protein kinase Fyn	P06241	Physical	CD2AP	No
ABL1	Abelson murine leukemia viral oncogene homolog 1	P00519	Physical	CD2AP	No
TPM1	Tropomyosin‐1	P09493	Genetic	COL1A1	Yes
FN1	Fibronectin	P02751	Physical	COL1A1	Yes
FYN	Tyrosine‐protein kinase Fyn	P06241	Genetic	COL2A1	No
FN1	Fibronectin	P02751	Physical	COL2A1	Yes
TPM1	Tropomyosin‐1	P09493	Genetic	CTNNB1	Yes
FYN	Tyrosine‐protein kinase Fyn	P06241	Physical	CTNNB1	No
FN1	Fibronectin	P02751	Genetic	CTNNB1	Yes
ABL1	Abelson murine leukemia viral oncogene homolog 1	P00519	Physical	CTNNB1	No
TRPC1	Short transient receptor potential channel 1	P48995	Genetic	DIAPH1	No
CD2AP	CD2‐associated protein	Q9Y5K6	Genetic	DIAPH1	Yes
ABL1	Abelson murine leukemia viral oncogene homolog 1	P00519	Genetic	DIAPH1	No
TLN1	Talin‐1	Q9Y490	Genetic	FYN	Yes
SIAH1	E3 Ub Ligase SIAH1 (seven in absentia homolog 1)	Q8IUQ4	Genetic	FYN	No
ABL1	Abelson murine leukemia viral oncogene homolog 1	P00519	Physical	FYN	No
TPM1	Tropomyosin‐1	P09493	Genetic	PACS1	Yes
CTNNB1	Beta Catenin	P35222	Genetic	PACS1	Yes
FN1	Fibronectin	P02751	Genetic	PACS1	Yes
ABL1	Abelson murine leukemia viral oncogene homolog 1	P00519	Genetic	PACS2	No
SIAH1	E3 Ub Ligase SIAH1 (seven in absentia homolog 1)	Q8IUQ4	Genetic	RGS7	No
CD2AP	CD2‐associated protein	Q9Y5K6	Genetic	RGS7	Yes
FN1	Fibronectin	P02751	Genetic	RGS7	Yes
ABL1	Abelson murine leukemia viral oncogene homolog 1	P00519	Genetic	RGS7	No
FN1	Fibronectin	P02751	Physical	TLN1	Yes
FN1	Fibronectin	P02751	Genetic	TLN1	Yes
TLN1	Talin‐1	Q9Y490	Genetic	TPM1	Yes
FN1	Fibronectin	P02751	Genetic	TPM1	Yes
FYN	Tyrosine‐protein kinase Fyn	P06241	Genetic	TRPC1	No

## Discussion

The highly conserved exocyst complex is classically known for its role in targeting and docking vesicles, carrying proteins, from the trans‐Golgi network (TGN) to the cell membrane (Lipschutz and Mostov 2002). We previously showed that the exocyst is necessary for ciliogenesis (Zuo et al. 2009). However, if the exocyst was only trafficking vesicles carrying proteins necessary for ciliogenesis, one would expect to find it just at the base of the primary cilium; instead, we show here that exocyst Sec10, an essential component of the exocyst complex (Zuo et al. 2009), localizes not only to the base of primary cilia, but also all along the primary cilium and, indeed, in what appear to be cilia‐interacting exosomes (Fig. 1). We also found that all eight members of the exocyst complex (Fig. 2), as well as many regulatory GTPases (Fig. 3, Table 1), are present in ELVs. Additionally, we show that fluorescently labeled exosomes appear to interact with renal cilia in *living* MDCK cells (Fig. 4, Movie S1). Lastly, we found that ELVs carry many known interactors of Smoothened (Fig. 4B, Table 2), Polycystins 1 and 2, and Fibrocystin (Fig. 4C, Table 3).

The exocyst has been shown to be regulated by many different small GTPases (Lipschutz and Mostov 2002) and to have other functions besides vesicle delivery (Lipschutz et al. 2003). The fact that the exocyst is found throughout the primary cilium suggests that it may have another role in the cilium besides simply trafficking vesicles carrying ciliary proteins from the TGN. The ciliary membrane is physically restricted from the adjacent cell membrane, and has a different protein and lipid composition than the adjacent cell membrane as well (Nachury et al. 2010). Therefore, one can imagine a scenario whereby the exocyst complex specifically targets ELVs containing ciliary proteins to the ciliary surface, or to multivesicular bodies (MVBs), for ELVs to be released into the extracellular environment, where they are subsequently taken up by primary cilia of the same or adjacent cells, and/or participate in ELV retrieval. This would be a logical mechanism to (Admyre et al. 2007) allow for the transfer of different membrane components to a physically restricted nascent ciliary membrane during ciliogenesis, to (Allen 1965) allow for the replenishment of various components of the ciliary membrane (including membrane proteins and, perhaps, different membrane lipids) in mature cilia, or (Bakeberg et al. 2011) to allow for intracellular (or intercellular) communication and signaling.

Further supporting a role other than simply targeting vesicles to the cilium, the entire exocyst, as well as many of its regulatory GTPases, are present in ELVs. This finding suggests that the exocyst complex is *actively regulated within* ELVs. Given that cells are highly efficient machines, we hypothesize that cells would not waste the energy required to target these proteins to ELVs if they were not serving a specific function within these vesicles. We propose that, in addition to being involved in targeting vesicles carrying proteins, the exocyst complex is centrally involved in regulating cilia/ELV interactions. Given the highly dynamic nature of cilia/exosome interactions observed in living cells (Fig. 4), we think that cilia are equally likely to be involved in the uptake (as suggested above) and/or secretion of ELVs. The exocyst could play a role in either or both of these processes. For instance, the exocyst could help dock and unload critical cargo from ELVs to the primary cilium after ELVs have bound to the cilium. Additionally, perhaps in a similar fashion as it functions in the cell membrane, instead of being involved in facilitating cargo unloading to the cilium (or in addition to this role), once inside a primary cilium, the exocyst complex could help target specific cargo to ELVs at the tip of the cilium for secretion.

Importantly, there is experimental evidence from different model systems that argues in favor of primary cilia actively secreting ELVs. For example, recent work from Wood et al. (2013) shows that the algae *Chlamydomonas* secretes *bioactive*, membrane‐bound vesicles from the distal ends of flagella, which are in many ways identical to cilia. In this case, the vesicles contain enzymes essential for budding. Although, one might be tempted to argue that this process could be limited to unicellular organisms, there is also experimental evidence of ciliary secretion in multicellular organisms. For example, as previously mentioned, Wang and colleagues recently demonstrated a role for extracellular vesicles carrying ciliary proteins in regulating mating behavior in *C. elegans* (Wang et al. 2014). Additionally, they showed that, in *C. elegans*, secretion of these vesicles does not take place via fusion of MVBs, but via vesicular budding at the ciliary base (Wang et al. 2014), thus providing an additional model system where the secretion of biologically active exosomes is associated with primary cilia. This leads to the hypothesis that these cilia‐associated mechanisms for secretion of bioactive exosomes are conserved in mammals.

Consistent with this hypothesis, hints of ciliary secretion in mammalian cells can be found in ciliary literature dating as far back as 1967 when *The Renewal of Photoreceptor Outer Segments* was first described (Young 1967). The outer segments (OS) of photoreceptor cells (which are, in fact, modified primary cilia) (Robertis 1956; Allen 1965), are renewed every 10 days (10% each day) by a process that involves the shedding of membrane‐bound discs/vesicles at the distal end of the photoreceptor/cilium (Sung and Chuang 2010). These vesicles are subsequently taken up and phagocytosed by the adjacent retinal pigment epithelial (RPE) cells (Young and Bok 1969; Bok and Hall 1971). Although this phenomenon was initially perceived as an eye‐specific process required for the maintenance of photoreceptor cells (Boesze‐Battaglia and Goldberg 2002; Sung and Chuang 2010), it clearly shows that at least some types of mammalian primary cilia secrete membrane‐bound vesicles from their distal ends, and, thus, suggests that secretion of exosomes by primary cilia is an evolutionarily conserved process maintained in mammals.

More recently, evidence suggests that cilia in other types of cells may also secrete membrane‐bound vesicles that mediate autocrine and paracrine signaling, and influence cellular (and likely bodily) functions. For example, Masyuk et al. (2010) demonstrated that biliary exosomes interact with primary cilia in cholangiocytes, and that this interaction, in turn influenced the expression of an miRNA that regulates cholangiocyte proliferation, as well as played a key role in keeping the MAPK signaling pathway quiescent. There are, therefore, several examples, in addition to *Chlamydomonas*, where interactions between cilia and secreted membrane vesicles can regulate cellular function.

In summary, we show that (Admyre et al. 2007) the exocyst localizes along the length of the primary cilium (Zuo et al. 2009) and in *vesicles that interact with the ciliary surface* (Fig. 1, insets); (Allen 1965) that all eight known members of the exocyst complex (Fig. 2) and (Bakeberg et al. 2011) most of its mammalian regulatory GTPases are found within ELVs (Fig. 3, Table 1); (Barr and Sternberg 1999) that renal cells interact with vesicles tagged with a fluorescently labeled ciliary membrane protein *in living cells* (Fig. 4), suggesting that cilia may be involved in the secretion and/or retrieval of ELVs; and that (Boesze‐Battaglia and Goldberg 2002;) ELVs carry a number of proteins known to interact with the ciliary proteins Smoothened, Polycystins 1 and 2 and Fibrocystin. These data, combined with the growing experimental evidence suggesting a biologically relevant role for cilia/ELV interactions, supports a model where the exocyst is centrally involved in the regulation of cilia/ELV interactions. The fact that the exocyst complex is required for normal ciliogenesis (Zuo et al. 2009), and that an exocyst mutation is the cause of the Joubert nephronophthisis form of PKD in a human family (Dixon‐Salazar et al. 2012), demonstrates the central role of the exocyst in regulating normal and pathogenic ciliogenesis, which now may also include cilia/ELV interactions.

## Acknowledgments

The University of Pennsylvania Biomedical Imaging Core Facility of the Cancer Center is gratefully acknowledged for providing imaging services.

## Conflict of Interest

None declared.

## Supplementary Material

**Movie S1**. MDCK cells stably expressing YFP-tagged Smoothened were grown on Transwell filters as described in the Methods section. Spinning disk confocal microscopy was used to visualize the cilia and Smoothened-YFP in living cells. Similar results were found using TIRF microscopy (not shown).
